# Feasibility of anticancer treatment using scalp cooling for patients with gynecological cancer in Japan: A case series study

**DOI:** 10.1111/jog.16270

**Published:** 2025-03-16

**Authors:** Shinya Oki, Yoko Ishihara, Sari Takahashi, Tomoyo Kato, Kana Ietani, Takeshi Makabe, Mizuki Kurihara, Akiko Ohno, Yoshiko Mikami, Hiroshi Yamashita

**Affiliations:** ^1^ Department of Obstetrics and Gynecology NHO Tokyo Medical Center Tokyo Japan; ^2^ Department of Obstetrics and Gynecology Keio University School of Medicine Tokyo Japan

**Keywords:** alopecia, gynecological cancer, Japanese, net promoter score, scalp cooling

## Abstract

**Aim:**

To evaluate the safety and efficacy of scalp cooling in preventing chemotherapy‐induced alopecia among Japanese patients with gynecological cancer.

**Methods:**

A retrospective study was conducted involving 16 patients with gynecological cancer who underwent chemotherapy with scalp cooling at our institution between January 2021 and April 2024. The completion rate of the planned regimens, the success rate (defined as hair loss ≤50%), hair volume recovery 8–12 weeks after chemotherapy, and adverse events were assessed. Additionally, patient satisfaction was measured using the net promoter score (NPS) following chemotherapy completion.

**Results:**

Of the 16 patients included in the study, chemotherapy regimens comprised six courses of combination therapy with paclitaxel plus carboplatin in 11 cases, three courses of the same regimen in three cases, and three courses of combination therapy with docetaxel plus carboplatin in two cases. The overall completion rate of the planned regimens was 75% (12/16 cases). Among the 12 cases, nine were evaluable for alopecia, with a success rate of 33.3%. The proportion of the patients who recovered hair volume from ≤50% to >50% was 83.3% in the occipital region. Adverse events were reported as follows: chills in 75.0%, jaw pain in 68.8%, headache in 31.3%, nausea in 18.8%, and hypertension and hunger in 12.5% each. The NPS for scalp cooling was 26.7 points.

**Conclusion:**

Scalp cooling is effective and safe in preventing hair loss and restoring hair volume in Japanese patients with gynecological cancer, suggesting high patient satisfaction with this treatment.

## INTRODUCTION

Gynecological cancers are among the most common malignancies in Japan. According to 2020 statistics, the annual number of new cases was 10 353 for cervical cancer, 17 779 for endometrial cancer, and 12 738 for ovarian cancer.^1^ These cancers collectively rank as the fourth most common female cancers in Japan, following breast, colorectal, and lung cancers. The incidence of cervical cancer begins to rise in women in their late 30s, while endometrial and ovarian cancers increase in women in their early 40s. These ages often coincide with significant responsibilities in work and family life, highlighting the broader societal impact of these diseases. Many patients with gynecologic cancers undergo taxane‐based chemotherapy, either pre‐ or postoperatively.

Common regimens, such as combination therapy with paclitaxel plus carboplatin or docetaxel plus carboplatin, are associated with Grade 2 or higher alopecia in 70%–90% of patients,^2^, ^3^ according to the Common Terminology Criteria for Adverse Events (CTCAE) Version 5.0. Chemotherapy‐induced alopecia results from damage to the rapidly dividing hair matrix cells, as >90% of scalp hair is in the anagen (growth) phase, where cell division is vigorous, making it particularly vulnerable during chemotherapy. Hair loss is a chemotherapy side effect that significantly impacts patients' quality of life (QOL).^4^ In a survey including 1853 patients with breast cancer, alopecia was identified as the most distressing chemotherapy‐related symptom.^5^ The first trial to prevent chemotherapy‐induced alopecia by cooling the scalp to reduce blood flow during chemotherapy in patients with breast cancer was reported in 1973.^6^ Since then, improvements in protocols and devices have led to better outcomes. For example, the Duncool Cap, marketed in the 1980s, could maintain scalp temperatures of 12–21°C for 1–3 h when cooled to −20°C and effectively reduce alopecia.^7^ However, the need for multiple replacements during prolonged chemotherapy prompted the development of more stable scalp‐cooling devices, now commercialized in Europe and the USA. In Japan, the PAXMAN system received regulatory approval in March 2019 as a medical device for preventing chemotherapy‐induced alopecia in all solid tumors. A domestic clinical trial in patients with breast cancer showed that 26.6% of patients treated with anthracycline‐ and taxane‐based regimens prevented alopecia at Grade 1 or below.^8^ Additionally, the restoration of hair volume at 12 weeks after completing chemotherapy was significantly higher in the scalp‐cooling group. Despite these advancements, most efforts to prevent chemotherapy‐induced alopecia have primarily focused on patients with breast cancer. Patients with gynecologic cancer in Japan have largely been supported by recommending wigs or hats, which come with challenges such as sweating during outdoor activities, rain, strong winds, or travel. To our knowledge, there have been no reports in Japan on the experience of scalp‐cooling treatment for patients with gynecologic cancer. Hence, we aimed to report the efficacy and safety of scalp‐cooling treatment specifically targeting Japanese patients with gynecologic cancer.

## METHODS

### Patients and data collection

Sixteen patients with gynecological cancer who underwent chemotherapy with scalp cooling at NHO Tokyo Medical Center between January 2021 and April 2024 were enrolled. All patients received postoperative adjuvant chemotherapy. Patients undergoing preoperative chemotherapy or those with recurrent disease were excluded. Anticancer regimens consisted of three or six courses of combination therapy with paclitaxel plus carboplatin (Paclitaxel 180 mg/m^2^, Carboplatin AUC6) or docetaxel plus carboplatin (Docetaxel 70 mg/m^2^, Carboplatin AUC6) administered every 3 weeks.

Data were retrospectively collected on age, cancer type, stage, surgery date, type of anticancer drug, number of scheduled courses, number of courses during which scalp cooling was performed, and adverse events related to scalp cooling. A satisfaction survey using the Net Promoter Score (NPS) was conducted after the completion of chemotherapy. This study was conducted with the approval of the NHO Tokyo Medical Center Ethics Committee (approval number: R24‐04) and in accordance with the Declaration of Helsinki, and the rights and privacy of all participants were protected. Written informed consent was obtained from all patients.

### Scalp cooling treatment

The Paxman Cooling device, manufactured by Paxman Cooler Limited (Huddersfield, UK), was used for scalp cooling. Treatment began 30 minutes before paclitaxel or docetaxel administration and continued for at least 90 minutes after the infusion completion. The cap size that best fit the patient's head was selected before the first course of treatment, and the cap was applied following the manufacturer's instructions (https://www.cmi.co.jp/products/1963/).

### Evaluation of alopecia

Alopecia was evaluated using Dean's alopecia scale^9^ as outlined below. Patients with hair loss of Grades 0–2 after completion of chemotherapy were classified as successful cases of scalp cooling. Photographs of the parietal and occipital areas were taken before the first course of chemotherapy, after three or six chemotherapy courses, and at 8–12 weeks post‐chemotherapy. The success rate of scalp cooling and the rate of hair volume recovery (defined as recovery from Grades 3 or 4 to Grade 0, 1, or 2) were evaluated at 8–12 weeks after chemotherapy completion. Alopecia was assessed independently by three individuals, and the average score was used as the final assessment.

#### Dean's alopecia scale

Grade 0: no alopecia; Grade 1: <25% alopecia; Grade 2: 25%–50% alopecia, Grade 3: 50%–75% alopecia, Grade 4: >75% alopecia.

### Satisfaction evaluation

A satisfaction survey using a questionnaire was conducted at 12 weeks after the end of chemotherapy on 16 patients who had undergone at least one course of chemotherapy with scalp cooling. The NPS^10^ was calculated based on the survey results.

### Statistical analysis

The data are presented as proportions or means ± standard deviations using Bell curve for Excel (Social Survey Research Information, Tokyo, Japan).

## RESULTS

### Patient characteristics

**FIGURE 1:**
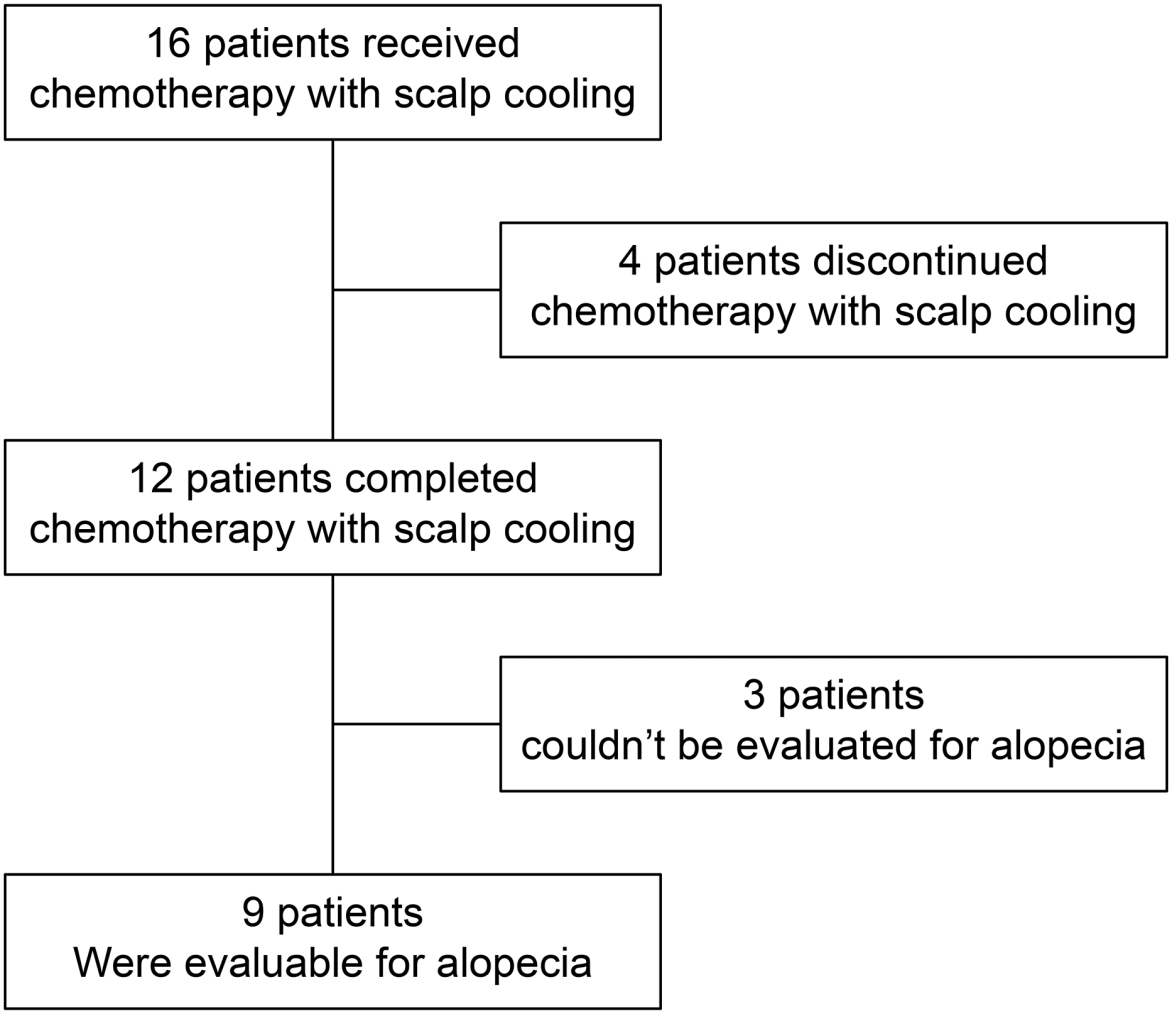
Flow diagram of the study.

The cohort included 13 patients with endometrial cancer and three patients with ovarian/fallopian tube cancer (median age, 60 [range, 39–82] years). All patients received postoperative adjuvant chemotherapy with scalp cooling. The planned regimens included six courses of combination therapy with paclitaxel plus carboplatin for 11 patients, three courses of combination therapy with paclitaxel plus carboplatin for three patients, and three courses of combination therapy with docetaxel plus carboplatin for two patients (Table 1). Details of the cases are presented in Table S1. The proportion of patients who completed the planned regimen under scalp cooling was 75% (12/16). Among the 12 patients who completed scalp cooling, nine were evaluable for alopecia prevention (Figure 1). The reasons for dropout included headache in one patient, hair loss in one patient, a change in chemotherapy regimen due to the development of hyperplastic exudative erythema in one patient, and termination of chemotherapy itself at the patient's request in one patient. The patients who dropped out of chemotherapy with scalp cooling due to headache and hair loss completed their planned chemotherapy courses without scalp cooling.

**TABLE 1:** Patient demographics.

Age (years)	Mean ± SD	60.9 ± 11 (*N* = 16)	
Type of cancer	Stage		
Endometrial cancer	I	62.5%	(10)
	II	12.5%	(2)
	III	6.3%	(1)
	IV	0%	(0)
Ovarina tumor, fallopian tube cancer	I	12.5%	(2)
	II	0%	(0)
	III	0%	(0)
	IV	6.3%	(1)
Chemotharapy regimen	TC^a^ × 6	68.5%	(11)
	TC × 3	18.8%	(3)
	DC^b^ × 3	12.5%	(2)

^a^ Paclitaxel 180 mg/m^2^ + Carboplatin 6 area under the curve (AUC) every 3 weeks.

^b^ Docetaxel 70 mg/m^2^ + Carboplatin 6 AUC every 3 weeks.

### Results of scalp‐cooling treatment (alopecia prevention effect)

Among the 12 patients who completed scalp cooling, nine were evaluable for its effect on the prevention of alopecia. Photographs of the occipital and parietal areas for these patients are shown in Figures S1–S9. The degree of alopecia after completing chemotherapy was as follows: Grade 0: 11.1% (one case), Grade 1: 11.1% (one case), Grade 2: 11.1% (one case), Grade 3: 33.3% (three cases), and Grade 4: 33.3% (three cases) in the occipital area. In the parietal area, the corresponding grades were as follows: Grade 0: 0% (zero cases), Grade 1: 22.2% (two cases), Grade 2: 11.1% (one case), Grade 3: 11.1% (one case), Grade 4: 33.3% (three cases), and Grade 4: 55.5% (three cases) (Table 2 and Table S2). The success rate of scalp cooling was 33.3% (3/9) for both the occipital and parietal areas.

**TABLE 2:** Evaluation of alopecia at the end of chemotherapy with scalp cooling.

Occipital area	No alopecia,^a^ *n* (%)		3 (33.3)			
	Alopecia grade^b^	Grade 0	Grade 1	Grade 2	Grade 3	Grade 4
	*n* (%)	1 (11.1)	1 (11.1)	1 (11.1)	3 (33.3)	3 (33.3)
	Example of head images	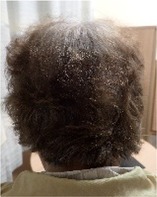	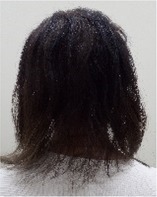	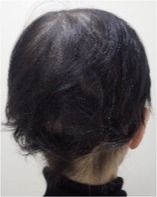	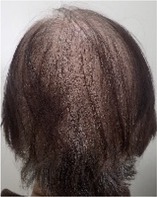	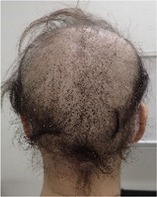
Parietal area	No alopecia,^a^ *n* (%)		3 (33.3)			
	Alopecia grade^b^	Grade 0	Grade 1	Grade 2	Grade 3	Grade 4
	*n* (%)	0 (0)	2 (22.2)	1 (11.1)	1 (11.1)	5 (55.5)
	Example of head images		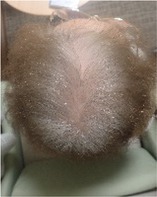	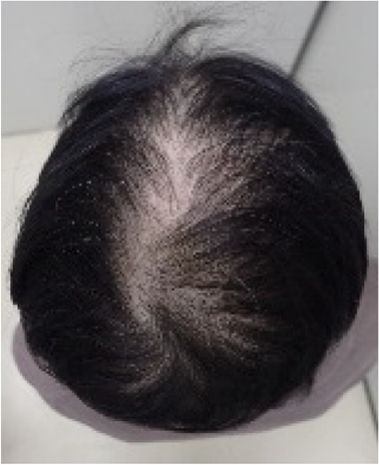	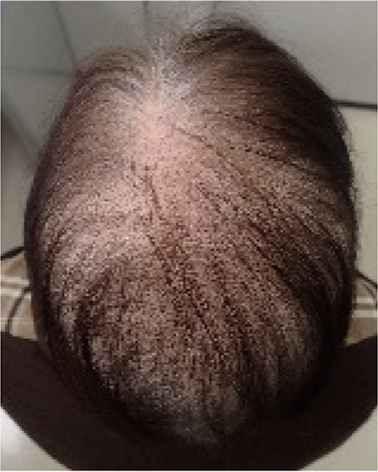	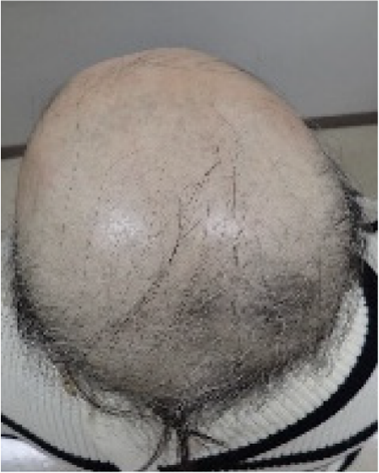

^a^ No case was defined as having Grade 0–2 alopecia.

^b^ Dean's alopecia scale was defined as follows: Grade 0: no alopecia; Grade 1: <25% alopecia; Grade 2: 25%–50% alopecia, Grade 3: 50%–75% alopecia, Grade 4: >75% alopecia.

### Results of scalp‐cooling treatment (hair volume recovery effect)

Photographs of the occipital and parietal areas were taken before the first course of chemotherapy, at the end of three or six courses of chemotherapy, and at 8–12 weeks after the end of chemotherapy (Figures S1–S9). The hair volume recovery effect at 8–12 weeks after chemotherapy completion is shown in Table 3. The percentage of patients with Grade 3–4 alopecia after chemotherapy completion who had recovered to Grade 0–2 alopecia at 8–12 weeks after completion of chemotherapy was 83.3% (5/6 patients) for the occipital area and 50% (3/6 patients) for the parietal area.

**TABLE 3:** Recovery from alopecia at 8 to 12 weeks after chemotherapy completion.

	Hair loss recovery at 8 to 12 weeks after the end of chemotherapy	*N* = 6, *n* (%)	Recovery rate^a^ (%)
Occipital area	Grade 3 or 4 → Grade 0	1 (16.7)	83.3
	Grade 3 or 4 → Grade 1	3 (50.0)	
	Grade 3 or 4 → Grade 2	1 (16.7)	
Parietal area	Grade 3 or 4 → Grade 0	1 (16.7)	50.0
	Grade 3 or 4 → Grade 1	1 (16.7)	
	Grade 3 or 4 → Grade 2	1 (16.7)	

^a^ The recovery rate was defined as the change in alopecia Grades 3 or 4 to Grade 0, 1, or 2 at 8–12 weeks after chemotherapy completion.

### Adverse events

During all observation periods, adverse events occurred in 87.5% (14/16) of patients, with 36 events related to scalp cooling (Table 4). These included chills in 75.0% (12/16), jaw pain due to strap tightening in 68.8% (11/16), headache in 31.3% (5/16), nausea in 18.8% (3/16), and hypertension and hunger in 12.5% (2/16) of cases. No scalp‐related skin diseases, including frostbite, were observed. All adverse events were resolved on the day of scalp cooling, and no serious adverse events were observed.

**TABLE 4:** Adverse events related to scalp cooling.

Adverse events	Numbers of case (*n* = 16)	Percentage
Chills	12	75.0
Jaw pain	11	68.8
Headache	5	31.1
Nausea	3	18.8
Hypertension	2	12.5
A sense of Hunger	2	12.5

### Net Promoter Score

**FIGURE 2:**
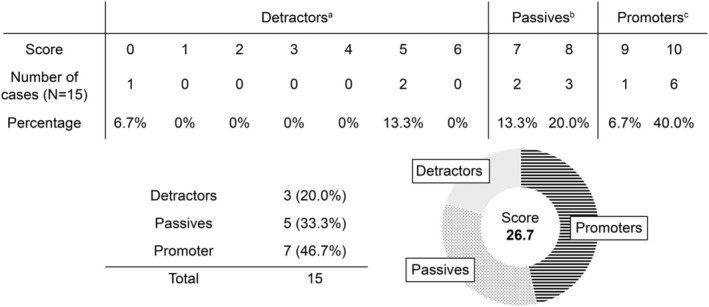
Net Promoter Score in patients with gynecological cancer who underwent chemotherapy with scalp cooling at least once. Patients who have received scalp cooling at least once were asked the question: “Would you recommend scalp cooling treatment to your family or acquaintances?” They were asked to rate their answers on a scale from 10 (highly recommend) to 0 (not recommend at all) points. The Net Promoter Score (NPS) is defined as the percentage of promoters (scores of 9 and 10 points) minus the percentage of detractors (scores of 0–6 points). ^a^Detractors are defined as patients who respond with a score of 0–6 points. ^b^Passives are defined as patients who respond with a score of 7–8 points. ^c^Promoters are defined as patients who respond with a score of 9–10 points.

A satisfaction survey conducted 12 weeks after chemotherapy had a response rate of 87.5% (14/16 cases) (Figure 2). The NPS for scalp cooling was 26.7 points.

## DISCUSSION

Combination therapy with paclitaxel plus carboplatin and docetaxel plus carboplatin, which are representative regimens of taxane‐based anticancer drugs in gynecological cancer, is associated with Grade 2 or higher alopecia in 70%–90% of patients.^2^, ^3^ Reports have indicated that the success rate of scalp cooling (defined as hair loss ≤50%) ranges from 26% to 56% in patients with early‐stage breast cancer receiving anthracycline‐ and taxane‐based regimens.^8^, ^11^, ^12^, ^13^ Additionally, hair volume recovery to <50% of the baseline level 12 weeks after chemotherapy was reported in 85%–100% of patients, compared with 50%–59% in those not receiving scalp cooling.^8^, ^13^ These findings suggest that scalp cooling not only prevents alopecia during chemotherapy but also promotes a high recovery rate of hair volume after treatment.

Recent studies have also shown significantly lower rates of chemotherapy‐induced persistent alopecia in patients with breast cancer (13.5%) at 6 months after completion of anthracycline‐ or taxane‐based chemotherapy with scalp cooling, compared with 52.0% in the control group.^14^ Regarding the site of alopecia, Kinoshita et al.^8^ conducted a detailed analysis and reported that 46.7% of hairs directly above the head exhibited 1/4 hair loss by the end of chemotherapy, a proportion slightly higher than that observed at other sites (the head was analyzed in five directions: front, right, left, rear, and directly above). Against this backdrop, scalp cooling for patients with breast cancer has been introduced at >100 facilities in Japan. In some facilities, >40% of patients receiving anticancer agents after surgery utilize scalp cooling, highlighting its importance in hair loss prevention. Given that paclitaxel and docetaxel—both taxane‐based anticancer drugs—are commonly used in treating gynecological and breast cancers, scalp cooling is also expected to be effective for patients with gynecological cancer. However, paclitaxel regimens for gynecological cancer differ from those of breast cancer in terms of dosage, administration time, intervals, and the number of cycles. For gynecological cancer, paclitaxel is administered at a dose of 175–180 mg/m^2^ over 3 h every 3 weeks for six cycles. In contrast, breast cancer regimens vary widely, with reported dosages of 80–90 mg/m^2^ over 1 h weekly for 12 cycles or 175 mg/m^2^ over 3 h every 2 or 3 weeks for four cycles.^11^, ^12^, ^13^ Therefore, the scalp cooling time for patients with gynecological cancer is approximately 2 h longer than that for patients with breast cancer. Despite these differences, no comprehensive analysis of scalp cooling for gynecological cancer or reports focusing on Japanese patients has been conducted until now.

A pilot study^14^ on scalp cooling in patients with gynecological cancer treated with paclitaxel plus carboplatin therapy and dose‐dense paclitaxel and carboplatin therapy reported mixed results. In the study, no effect of scalp cooling was observed for combinations of paclitaxel plus carboplatin, and the success rate of scalp cooling in dose‐dense paclitaxel and carboplatin therapy was 80%.^15^ However, this was a relatively small study (eight patients in the combination with paclitaxel plus carboplatin therapy group and 20 patients in the dose‐dense paclitaxel and carboplatin therapy group). Among the group treated with a combination of paclitaxel plus carboplatin, hair loss could be evaluated in only five cases and no follow‐up study was conducted to assess subsequent recovery of hair volume. Conversely, when the combination with paclitaxel plus carboplatin therapy was combined with scalp cooling, regardless of cancer type, it was reported that 38% of patients did not need head covering including using a wig^16^ and 73% of patients that underwent scalp cooling had hair loss controlled to ≤25%.^17^ These findings suggested that scalp cooling for patients with gynecological cancer is expected to have a certain therapeutic effect. However, the number of facilities in Japan that offer scalp cooling for these patients is currently limited to a few. This study represents the first report in Japan examining scalp cooling for gynecological cancer patients. It retrospectively analyzed its efficacy in preventing hair loss, restoring hair volume 8–12 weeks after completion of chemotherapy, and associated adverse events. Unlike previous studies that focused on the effectiveness and QOL outcomes of scalp cooling, this study also assessed patient satisfaction through a survey. In our study, the success rate of scalp‐cooling treatment was 33.3%, while the recovery rate of hair volume at 8–12 weeks after the end of chemotherapy was 83.3% for the occipital area and 50% for the parietal area. These results were similar to those reported by Kinoshita et al.^8^ in the same Japanese cohort, although the cancer types were different. Concerning the rate of hair volume recovery, the parietal area was slightly lower, which may be due in part to the wide range of time points at which hair volume recovery was evaluated, ranging from 8 to 12 weeks. Regarding adverse events related to scalp cooling treatment, the administration time of paclitaxel to patients with gynecological cancer is 3 h, which is 2 h longer than that of taxane anticancer drugs for patients with breast cancer. The total duration of scalp cooling was 5 h, but the frequency of chills and jaw pain in this study was 68–75%, similar to that reported by Kinoshita et al.^8^ In contrast, 12.5% of the patients complained of hunger due to the inability to tolerate orally after the straps were tightened for a long time, which was considered to be an adverse event specific to scalp cooling in patients with gynecological cancer. Patient satisfaction with scalp cooling was assessed using the NPS, a “customer loyalty” index proposed by Frederick F. Reichheld, author of *The One Number You Need to Grow*. The NPS asks a simple question: “Would you recommend this product or service to your family or acquaintances?” A respondent is considered a promoter only if they are genuinely willing to recommend the product or service to their family and acquaintances. Many global companies use this metric as a predictive indicator of whether a product or service will be widely accepted in the future, in contrast to traditional customer satisfaction surveys, which often reflect personal perceptions. In Japan, many products or services tend to receive negative NPS scores^18^ because Japanese respondents often prefer moderate responses and avoid extreme ones.^19^ However, the NPS score for the scalp cooling treatment conducted in our study was 26.7 points. Additionally, 50% of the respondents were promoters, with some of these promoters being patients who experienced Grade 3 or higher hair loss. This suggests that patient satisfaction with scalp cooling may be influenced not only by the degree of hair loss but also by the recovery of hair volume and other factors. A more detailed analysis of these results could provide insights into enhancing patient satisfaction with scalp cooling treatment.

A limitation of this study is its retrospective design, which may introduce bias due to the absence of randomization and control groups. Moreover, the relatively small sample size limits the generalizability of the findings. The variation in chemotherapy regimens and the lack of standardized follow‐up assessments for hair recovery further constrain the study's conclusions.

In conclusion, scalp cooling for patients with gynecological cancer can be safely administered, effectively preventing hair loss and promoting hair volume recovery. It is also associated with high patient satisfaction, suggesting that it may become an important supportive treatment in managing chemotherapy‐induced alopecia. Further studies are warranted to validate these findings and optimize treatment protocols.

## AUTHOR CONTRIBUTIONS

**Shinya Oki:** Conceptualization; data curation; formal analysis; methodology; project administration; writing – original draft. **Yoko Ishihara:** Data curation. **Sari Takahashi:** Data curation. **Tomoyo Kato:** Data curation. **Kana Ietani:** Writing – review and editing. **Takeshi Makabe:** Writing – review and editing. **Mizuki Kurihara:** Writing – review and editing. **Akiko Ohno:** Writing – review and editing. **Yoshiko Mikami:** Writing – review and editing. **Hiroshi Yamashita:** Supervision; writing – review and editing.

## CONFLICT OF INTEREST STATEMENT

The authors declare no conflict of interest for this article.

## Supporting information


**Data S1.** Photographs of the parietal and occipital areas in Cases 3, 5, 9, 11, 12, 13, 14, 15, and 16 before the first course of chemotherapy, at the end of three or six courses of chemotherapy, and at 8–12 weeks after chemotherapy completion. Evaluation of alopecia according to Dean's alopecia scale are shown below each photograph.

**Table S1.** Characteristics of the 16 patients who underwent chemotherapy with scalp cooling for at least one course.
**Table S2.** Evaluation of alopecia according to Dean's alopecia scale.

## Data Availability

The data that supports the findings of this study are available in the supplementary material of this article.
