# Supplementary figures and images for: Feasibility of anticancer treatment using scalp cooling for patients with gynecological cancer in Japan: A case series study
Source: J Obstet Gynaecol Res. 2025 Mar 16;51(3):e16270. doi: 10.1111/jog.16270 (PMC11911022; doi:10.1111/jog.16270)

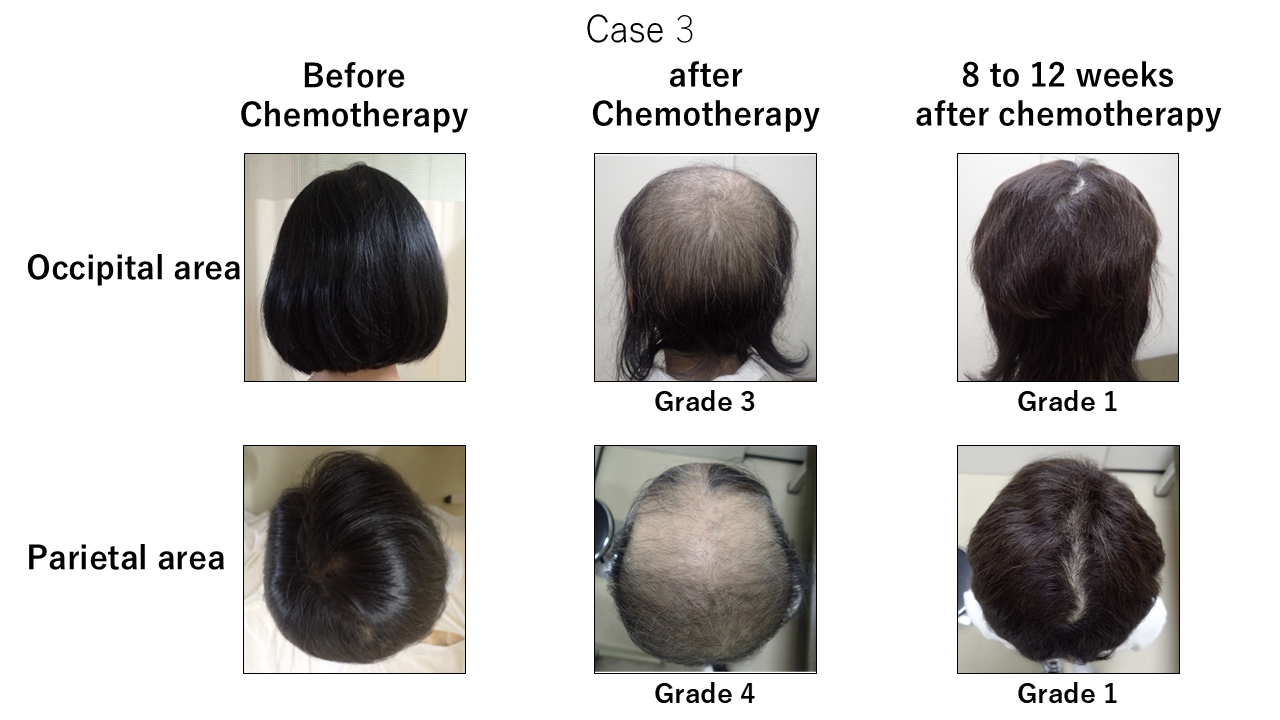

Supplement: Supplementary file 1 — Data S1. Photographs of the parietal and occipital areas in Cases 3, 5, 9, 11, 12, 13, 14, 15, and 16 before the first course of chemotherapy, at the end of three or six courses of chemotherapy, and at 8–12 weeks after chemotherapy completion. Evaluation of alopecia according to Dean's alopecia scale are shown below each photograph. [file JOG-51-0-s002.zip › jog16270-sup-0001-DataS1/jog16270-sup-0001-supinfo_Figure_S1.TIF]

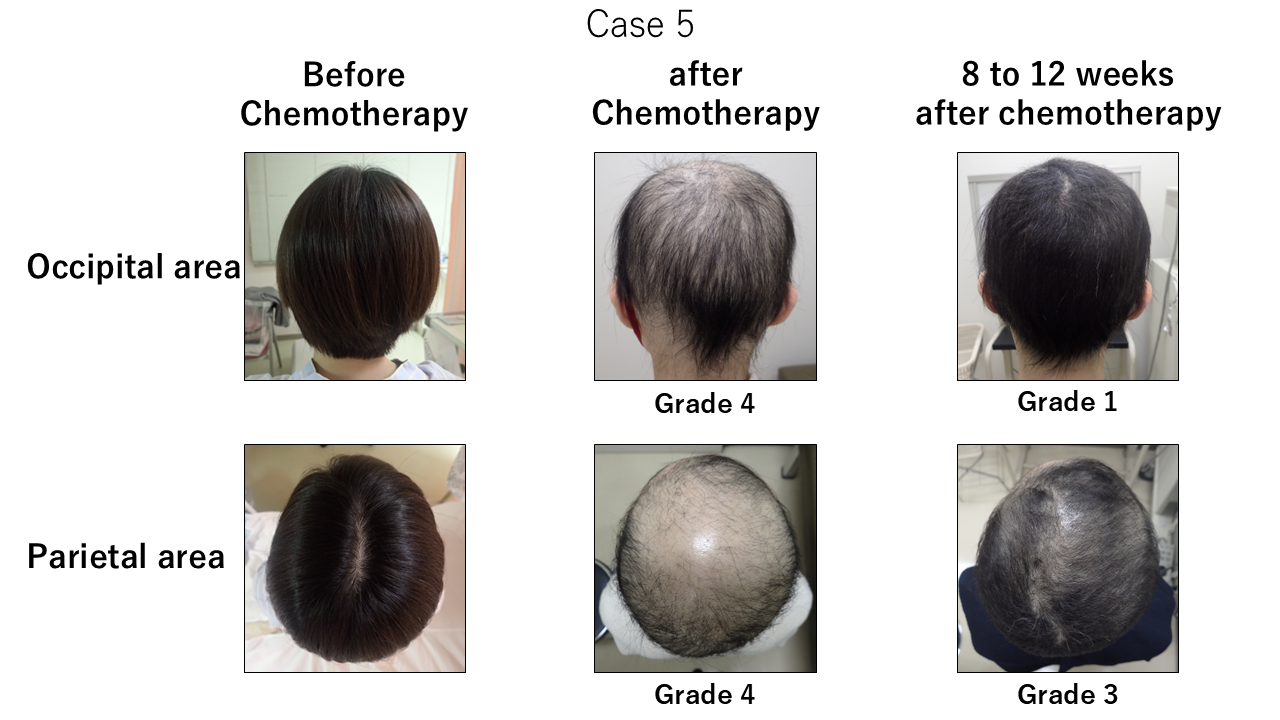

Supplement: Supplementary file 1 — Data S1. Photographs of the parietal and occipital areas in Cases 3, 5, 9, 11, 12, 13, 14, 15, and 16 before the first course of chemotherapy, at the end of three or six courses of chemotherapy, and at 8–12 weeks after chemotherapy completion. Evaluation of alopecia according to Dean's alopecia scale are shown below each photograph. [file JOG-51-0-s002.zip › jog16270-sup-0001-DataS1/jog16270-sup-0002-supinfo_Figure_S2.TIF]

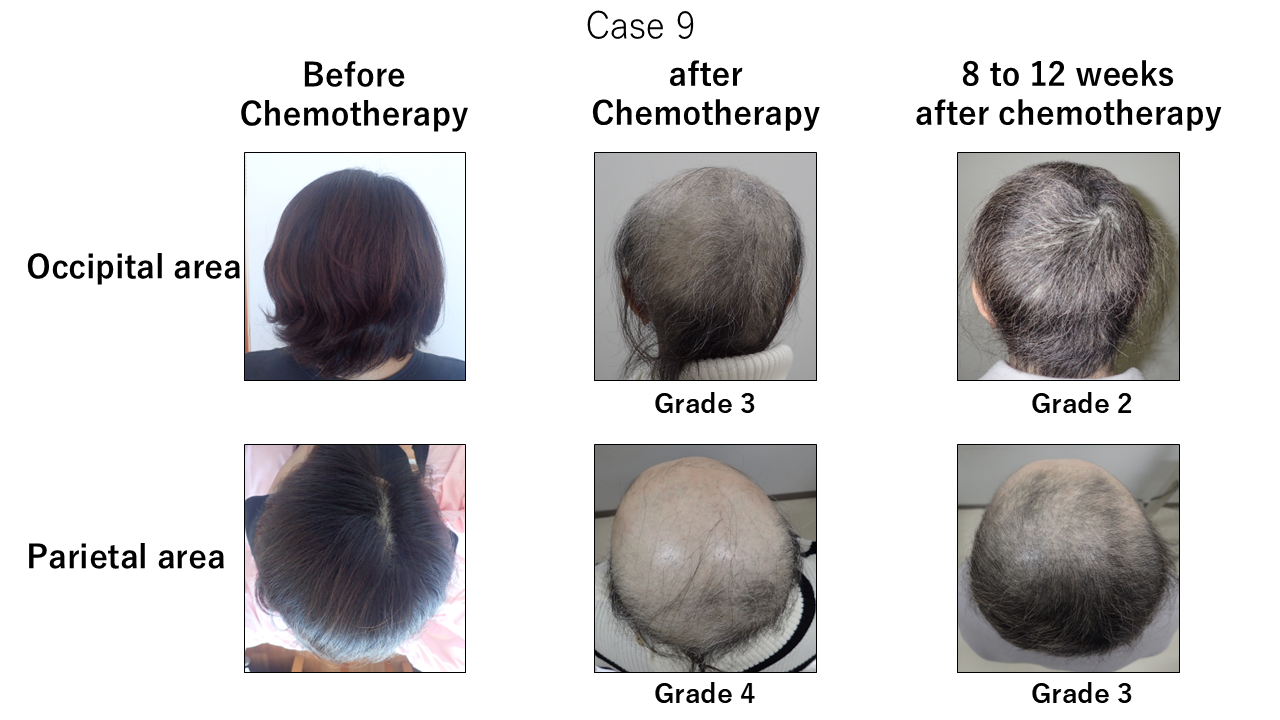

Supplement: Supplementary file 1 — Data S1. Photographs of the parietal and occipital areas in Cases 3, 5, 9, 11, 12, 13, 14, 15, and 16 before the first course of chemotherapy, at the end of three or six courses of chemotherapy, and at 8–12 weeks after chemotherapy completion. Evaluation of alopecia according to Dean's alopecia scale are shown below each photograph. [file JOG-51-0-s002.zip › jog16270-sup-0001-DataS1/jog16270-sup-0003-supinfo_Figure_S3.TIF]

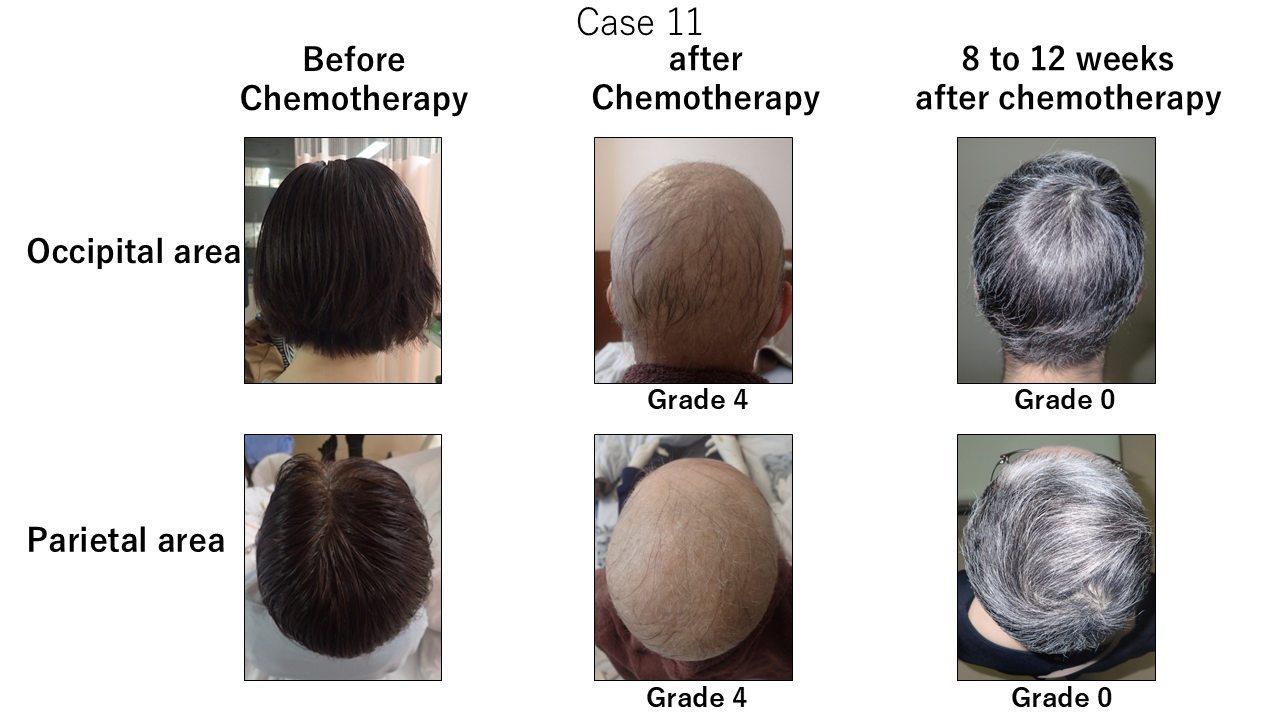

Supplement: Supplementary file 1 — Data S1. Photographs of the parietal and occipital areas in Cases 3, 5, 9, 11, 12, 13, 14, 15, and 16 before the first course of chemotherapy, at the end of three or six courses of chemotherapy, and at 8–12 weeks after chemotherapy completion. Evaluation of alopecia according to Dean's alopecia scale are shown below each photograph. [file JOG-51-0-s002.zip › jog16270-sup-0001-DataS1/jog16270-sup-0004-supinfo_Figure_S4.TIF]

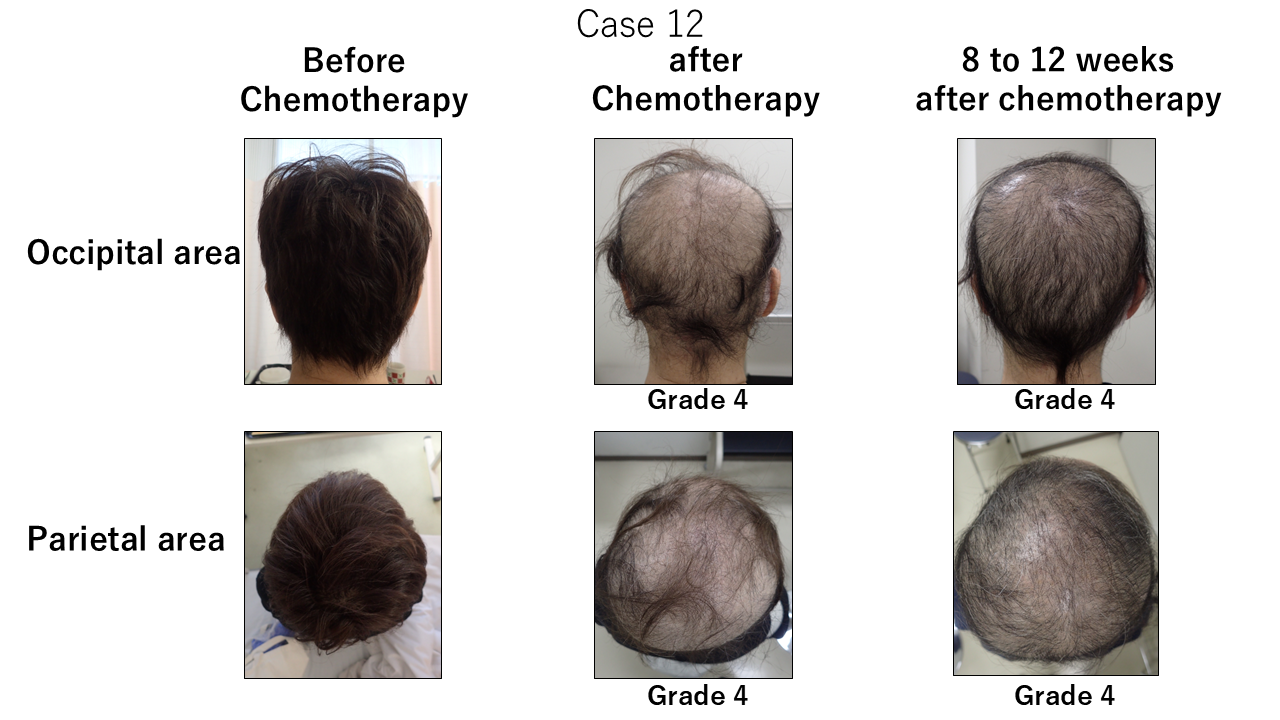

Supplement: Supplementary file 1 — Data S1. Photographs of the parietal and occipital areas in Cases 3, 5, 9, 11, 12, 13, 14, 15, and 16 before the first course of chemotherapy, at the end of three or six courses of chemotherapy, and at 8–12 weeks after chemotherapy completion. Evaluation of alopecia according to Dean's alopecia scale are shown below each photograph. [file JOG-51-0-s002.zip › jog16270-sup-0001-DataS1/jog16270-sup-0005-supinfo_Figure_S5.TIF]

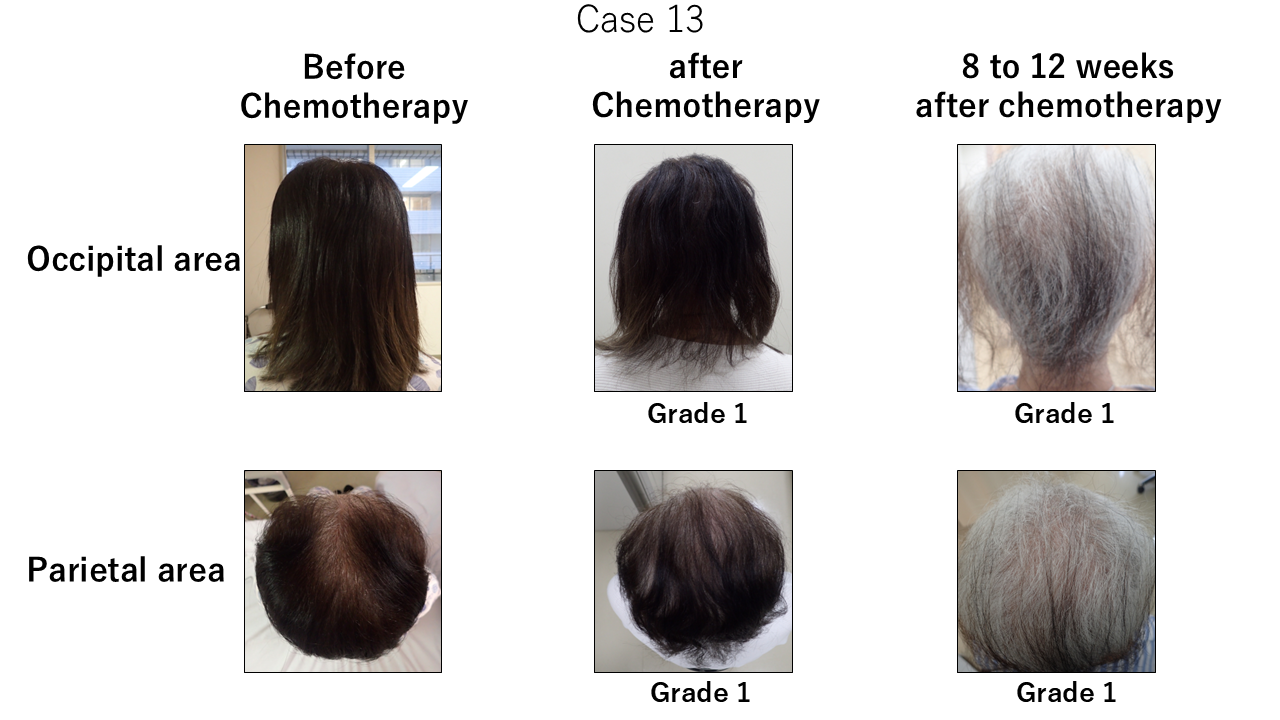

Supplement: Supplementary file 1 — Data S1. Photographs of the parietal and occipital areas in Cases 3, 5, 9, 11, 12, 13, 14, 15, and 16 before the first course of chemotherapy, at the end of three or six courses of chemotherapy, and at 8–12 weeks after chemotherapy completion. Evaluation of alopecia according to Dean's alopecia scale are shown below each photograph. [file JOG-51-0-s002.zip › jog16270-sup-0001-DataS1/jog16270-sup-0006-supinfo_Figure_S6.TIF]

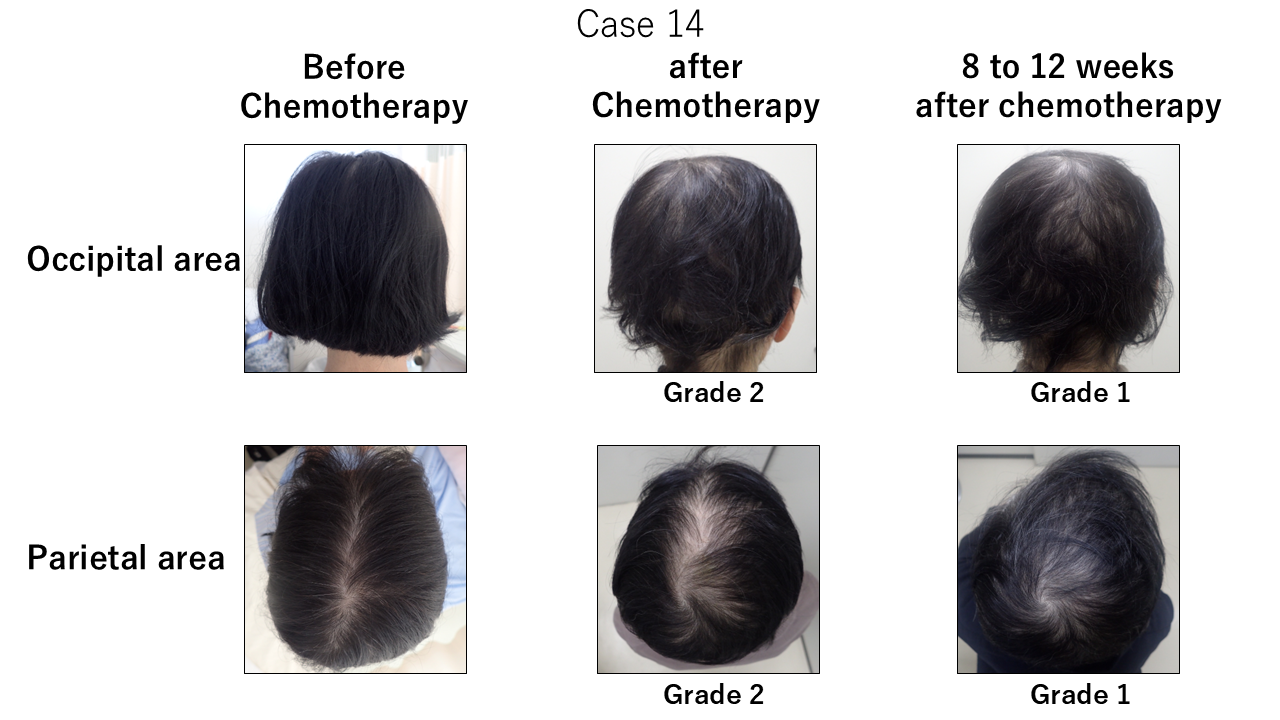

Supplement: Supplementary file 1 — Data S1. Photographs of the parietal and occipital areas in Cases 3, 5, 9, 11, 12, 13, 14, 15, and 16 before the first course of chemotherapy, at the end of three or six courses of chemotherapy, and at 8–12 weeks after chemotherapy completion. Evaluation of alopecia according to Dean's alopecia scale are shown below each photograph. [file JOG-51-0-s002.zip › jog16270-sup-0001-DataS1/jog16270-sup-0007-supinfo_Figure_S7.TIF]

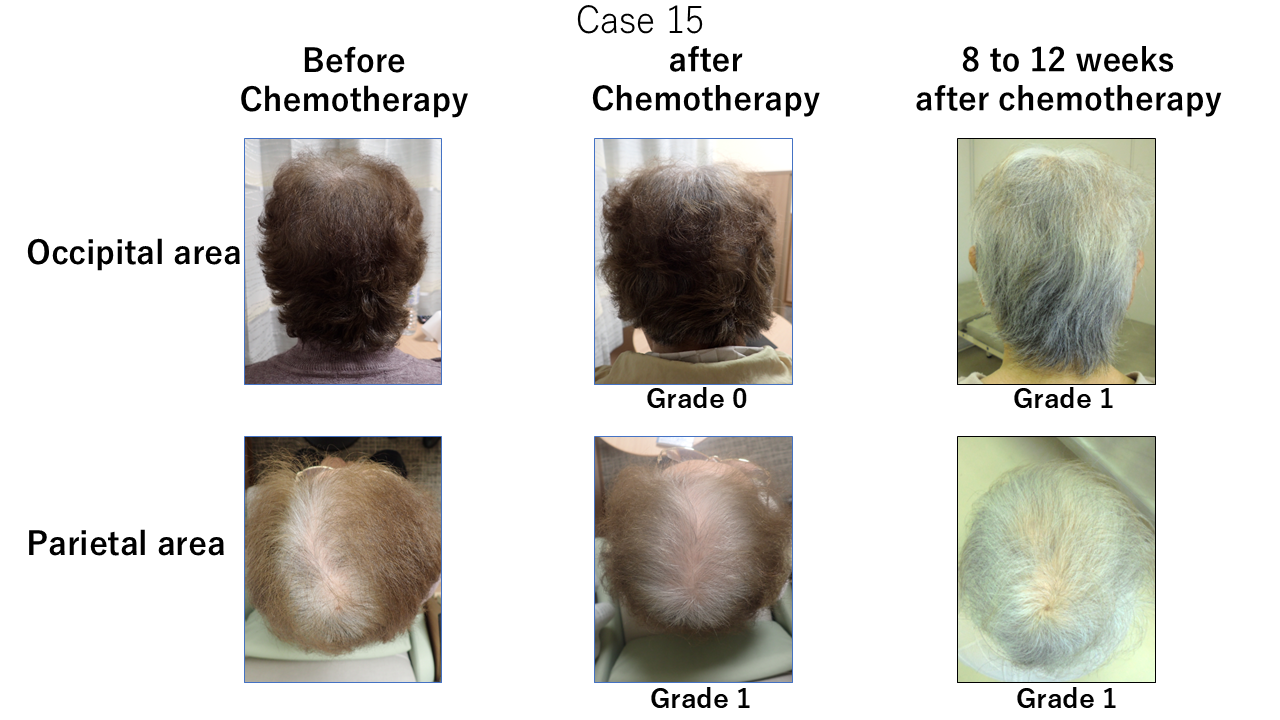

Supplement: Supplementary file 1 — Data S1. Photographs of the parietal and occipital areas in Cases 3, 5, 9, 11, 12, 13, 14, 15, and 16 before the first course of chemotherapy, at the end of three or six courses of chemotherapy, and at 8–12 weeks after chemotherapy completion. Evaluation of alopecia according to Dean's alopecia scale are shown below each photograph. [file JOG-51-0-s002.zip › jog16270-sup-0001-DataS1/jog16270-sup-0008-supinfo_Figure_S8.TIF]

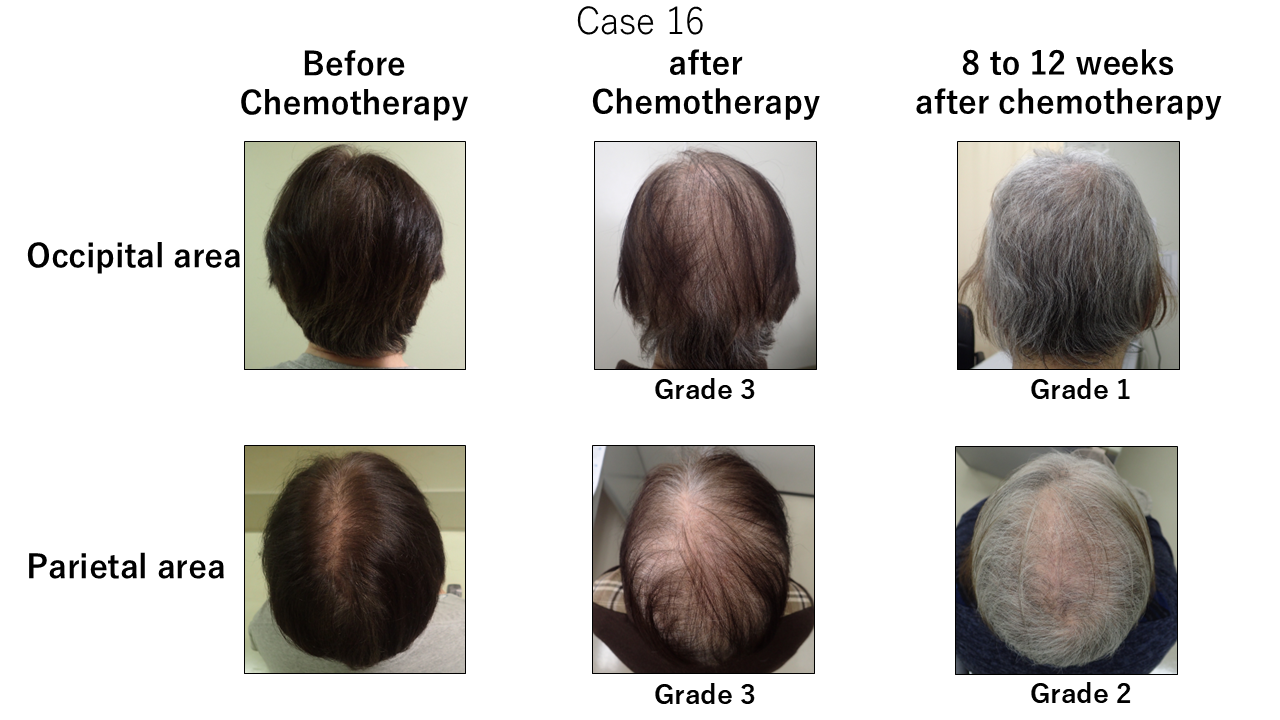

Supplement: Supplementary file 1 — Data S1. Photographs of the parietal and occipital areas in Cases 3, 5, 9, 11, 12, 13, 14, 15, and 16 before the first course of chemotherapy, at the end of three or six courses of chemotherapy, and at 8–12 weeks after chemotherapy completion. Evaluation of alopecia according to Dean's alopecia scale are shown below each photograph. [file JOG-51-0-s002.zip › jog16270-sup-0001-DataS1/jog16270-sup-0009-supinfo_Figure_S9.TIF]
