# Supplementary material for: Feasibility of anticancer treatment using scalp cooling for patients with gynecological cancer in Japan: A case series study
Source: J Obstet Gynaecol Res. 2025 Mar 16;51(3):e16270. doi: 10.1111/jog.16270 (PMC11911022; doi:10.1111/jog.16270)
Supplement: Supplementary file 2 — Table S1. Characteristics of the 16 patients who underwent chemotherapy with scalp cooling for at least one course. Table S2. Evaluation of alopecia according to Dean's alopecia scale. [file JOG-51-0-s001.docx]

**Supplementary Table 1. Characteristics of the 16 patients who underwent chemotherapy with scalp cooling for at least one course.**

| Case | Age  (years) | Type of cancer | Chemotherapy regimen | Scheduled number | number that actually underwent each course | Completion of chemotherapy with scalp cooling  Yes/ No | Alopecia evaluated  Yes/ No |
| --- | --- | --- | --- | --- | --- | --- | --- |
| 1 | 53 | Endometrial cancer | TC | 6 | 6 | Yes | No |
| 2 | 52 | Endometrial cancer | TC | 6 | 1 | No | No |
| 3 | 54 | Endometrial cancer | TC | 6 | 6 | Yes | Yes |
| 4 | 58 | Endometrial cancer | TC | 6 | 6 | Yes | No |
| 5 | 39 | Endometrial cancer | TC | 6 | 6 | Yes | Yes |
| 6 | 74 | Endometrial cancer | TC | 6 | 2 | No | No |
| 7 | 50 | Endometrial cancer | TC | 6 | 6 | Yes | No |
| 8 | 73 | Endometrial cancer | TC | 6 | 1 | No | No |
| 9 | 63 | Fallopian Tube Cancer | TC | 6 | 6 | Yes | Yes |
| 10 | 48 | Ovarian cancer | TC | 6 | 1 | No | No |
| 11 | 54 | Ovarian cancer | TC | 6 | 6 | Yes | Yes |
| 12 | 62 | Endometrial cancer | TC | 3 | 3 | Yes | Yes |
| 13 | 73 | Endometrial cancer | TC | 3 | 3 | Yes | Yes |
| 14 | 68 | Endometrial cancer | TC | 3 | 3 | Yes | Yes |
| 15 | 82 | Endometrial cancer | DC | 3 | 3 | Yes | Yes |
| 16 | 63 | Endometrial cancer | DC | 3 | 3 | Yes | Yes |

**Supplementary Table 2. Evaluation of alopecia according to Dean’s alopecia scale**

| Case | **Occipital area** | |  | **Parietal area** | |
| --- | --- | --- | --- | --- | --- |
|  | After completion of chemotherapy | 8 to 12 weeks after the end of chemotherapy |  | After the end of chemotherapy | 8 to 12 weeks after the end of chemotherapy |
| ３ | G3 | G1 |  | G4 | G1 |
| 5 | G4 | G1 |  | G4 | G3 |
| 9 | G3 | G2 |  | G4 | G3 |
| 11 | G4 | G0 |  | G4 | G0 |
| 12 | G4 | G4 |  | G4 | G4 |
| 13 | G1 | G1 |  | G1 | G1 |
| 14 | G2 | G1 |  | G2 | G1 |
| 15 | G0 | G1 |  | G1 | G1 |
| 16 | G3 | G1 |  | G3 | G2 |
